# Making a bat: The developmental basis of bat evolution

**DOI:** 10.1590/1678-4685-GMB-2019-0146

**Published:** 2021-02-08

**Authors:** Alexa Sadier, Daniel J. Urban, Neal Anthwal, Aidan O. Howenstine, Ishani Sinha, Karen E. Sears

**Affiliations:** 1 University of California at Los Angeles, Department of Ecology and Evolutionary Biology, Los Angeles, USA.; 2 American Museum of Natural History, Department of Mammalogy, New York, USA.

**Keywords:** Chiroptera, wing, craniofacial, sensory adaptations, teeth

## Abstract

Bats are incredibly diverse, both morphologically and taxonomically. Bats are the only mammalian group to have achieved powered flight, an adaptation that is hypothesized to have allowed them to colonize various and diverse ecological niches. However, the lack of fossils capturing the transition from terrestrial mammal to volant chiropteran has obscured much of our understanding of bat evolution. Over the last 20 years, the emergence of evo-devo in non-model species has started to fill this gap by uncovering some developmental mechanisms at the origin of bat diversification. In this review, we highlight key aspects of studies that have used bats as a model for morphological adaptations, diversification during adaptive radiations, and morphological novelty. To do so, we review current and ongoing studies on bat evolution. We first investigate morphological specialization by reviewing current knowledge about wing and face evolution. Then, we explore the mechanisms behind adaptive diversification in various ecological contexts using vision and dentition. Finally, we highlight the emerging work into morphological novelties using bat wing membranes.

## Introduction: Diversity and phylogeny of bats

Following the acquisition of powered flight, bats (Order Chiroptera) have diversified into ~1411 species that comprise ~20% of all mammals (Gunnell and Simmons, 2005; Burgin *et al.*, 2018). While chiropteran diversity is highest in the tropics (Shi *et al.*, 2018), bats are ubiquitous across the globe, with species inhabiting every continent except for Antarctica. Within this broad geographic distribution, bats occupy a wide array of ecological niches, with diets spanning insectivory, frugivory, nectarivory, piscivory, sanguinivory, and carnivory. This diverse range of feeding types makes bats important members of many ecosystems, where they act as pollinators, predators, and key regulators of insect populations (Hill and Smith, 1984). Bats’ diversity can also be observed in their unique morphologies and novel features; bats display greatly elongated digits, uniquely pliable bone structure, and high variance in wing membrane shape and craniofacial structure, among other unique characteristics (Hill and Smith, 1984; Dumont, 2007; Cooper and Sears, 2013). Bats also display a wide range of life spans (Wilkinson and South 2002) with at least four bat lineages exhibiting “extreme longevity” (Wilkinson and Adams, 2019) and unique immune systems (Jebb *et al.*, 2020). The wide array of specialized niches, morphologies and life spans that characterize bats makes them an excellent study system to investigate links between development and functional morphology, ecomorphology, adaptive radiations, and macroevolution as well as senescence, aging and the immune system.

**Figure 1 -:**
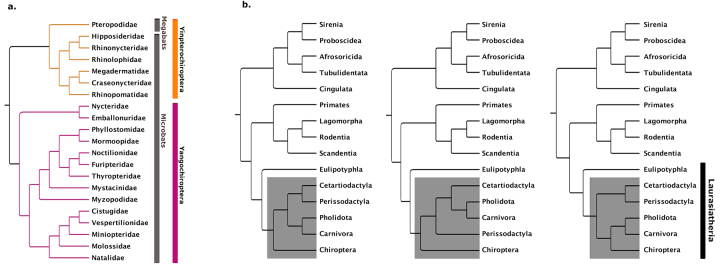
Phylogeny of bats. (a) Cladogram* showing intraordinal phylogenetic relationships in Chiroptera (adapted from Teeling *et al.*, 2018). Based on molecular data, Chiroptera is now classified into Yinpterochiroptera (orange) and Yangochiroptera (magenta), while the traditional classification into microbats and megabats is shown in grey. (b) Cladogram* showing the interordinal relationship between Chiroptera and other mammalian taxa (adapted from Jebb *et al.*, 2020). Three of the best supported tree topologies for the relationship between the Laurasiatherian lineages Carnivora, Perrissodactyla, Cetartiodactyla, Pholidota and Chiroptera (indicated by shaded area) are shown. *Branch lengths do not indicate distance.

Traditionally, bats have been classified as Microchiroptera (microbats) and Megachiroptera (megabats), but molecular evidence has revealed that microbats are not a reciprocally monophyletic clade (Teeling *et al.*, 2005). Bats have therefore been reclassified as Yinpterochiroptera and Yangochiroptera, resolving the paraphyly of Microchiroptera (Teeling *et al.*, 2005) (Figure 1a). Yinpterochiroptera consist of the lineage previously classified as megabats (Pteropodidae) and the microbat group Rhinolophidae, while Yangochiroptera consist of the remaining three microbat lineages Emballonuroidea, Noctilionoidea, and Vespertilonoidea (Teeling *et al.*, 2005). 

In relation to other mammals, molecular phylogenetic studies place bats within Laurasiatheria (Tsagkogeorga *et al.*, 2013) as sister to Cetartiodactyla, Pholidota, Carnivora and Perissodactyla, although the interordinal relationships between these sister clades remains unresolved (Jebb *et al.*, 2020) (Figure 1b). The ancestral relationship to Eulipotyphla (moles and shrews) as the basal clade within Laurasiatheria (Tsagkogeorga *et al.*, 2013; Jebb *et al.*, 2020), combined with the (limited) fossil data suggests that the ancestor to bats might have been a small, quadrupedal, insectivorous mammal with pawed limbs (Gunnell and Simmons, 2005). However, the patterns and processes of the evolutionary transition of this hypothetical ancestor into a bat remain speculative, in large part because this transition is largely uncaptured by the fossil record. Indeed, bat fossil records have the lowest skeletal completeness metrics among all tetrapods analyzed to date, with most fossils being isolated bat teeth (Brown *et al.*, 2019). The oldest known bat fossils date from the early Eocene, approximately 52.5 million years ago; these fossils already bear remarkable morphological similarities with extant bats and when alive the animals were likely capable of powered flight (Teeling *et al.*, 2000; Gunnell and Simmons, 2005; Simmons *et al.*, 2008). Due to this dearth of transitional paleontological information, many researchers have begun utilizing studies of bat development and phylogenetic relationships to better understand the origin of bats and how they came to occupy such a diverse array of niches. Examining the developmental processes that generate the tremendous amount of variation in this clade has the potential to provide insights into how macroevolutionary transitions occur and what processes facilitate the origins of variation. In this article, we explore some of the insights into the processes of morphological specialization of homologous structures, morphological diversification during adaptive radiations, and the origins of morphological novelty that research in bats have provided.

## Morphological specialization of homologous structures

Much of the embryological research to date using bats as model organisms has investigated the role of developmental changes in the morphological specialization of homologous structures, i.e., structures derived from a common ancestral structure. Here we discuss past and ongoing research on the developmental basis of the morphological specialization of the bat wing skeleton for flight, and of the diversification of bat cranial structures for many diets and behaviors.

## Bat wings: Extended outgrowth and forelimb lengthening

Given that bats are the only mammals capable of powered flight, it is perhaps not surprising that one of their most striking and recognizable features is their wings. The earliest bats preserved in the fossil record possess the defining characteristics of the bat wing: an overall enlarged forelimb, flight membranes, and elongated skeletal elements (Gunnell and Simmons, 2005; Simmons *et al.*, 2008). In fact, morphometric analysis of the forelimb elements of fossil and extant bats suggests that the relative length of bat forelimbs and digits has not significantly changed in 50 million years (Sears *et al.*, 2006). With clues to the origin of chiropteran wing structures currently lacking in the fossil record, some studies have turned to developmental mechanisms to try to understand this transition, often using comparisons of bat and mouse limb, the latter being a model for limb development (Cretekos *et al.*, 2008; Adams, 2008). 

At the time of the initial cartilaginous condensation of the digits, the future skeletal elements in bat and mice forelimb are similar in size (Sears *et al.*, 2006; Hockman *et al.*, 2009). However, rates of chondrocyte proliferation and differentiation soon increase notably in bat long bones, and the long bones increase in relative length (Sears *et al.*, 2006; Farnum *et al.*, 2008). The result is that the final relative size of the bat forelimb, including the digits, is larger than that of ancestral, terrestrial mammals. Studies of the allometry of bat bones support the hypothesis that differences in the growth rates of bat long bones help drive differences in long bone proportions among bat species (López‐Aguirre *et al.*, 2019).

**Figure 2 -:**
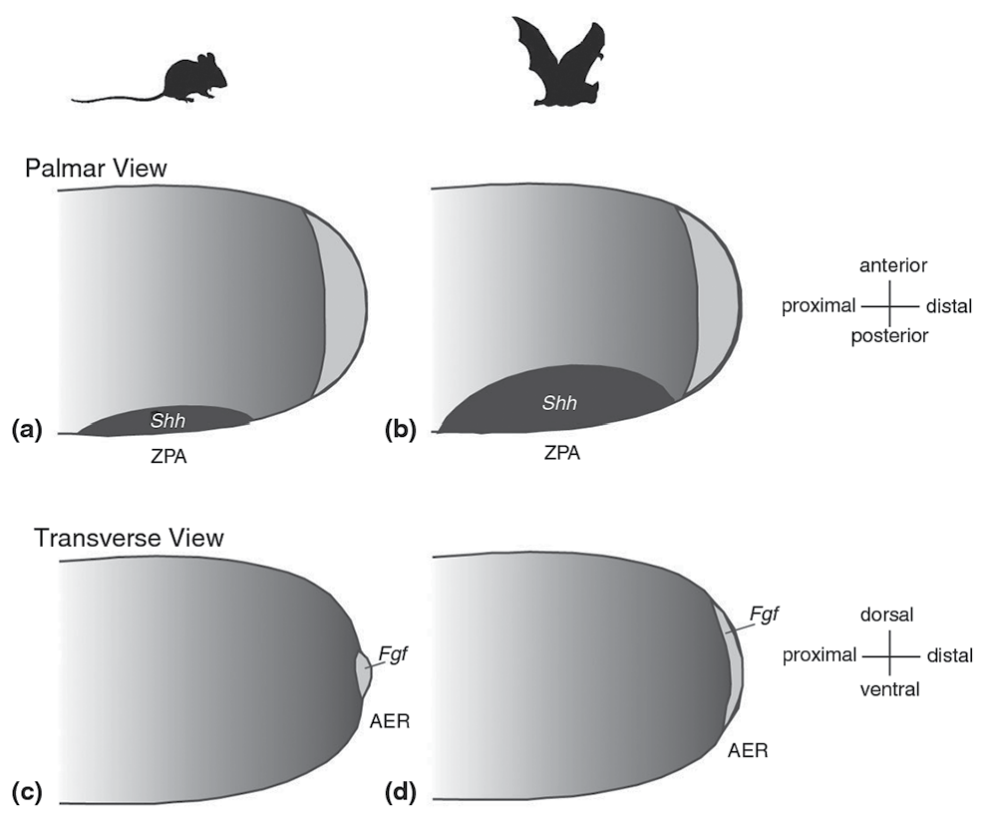
Differences in signaling centers between bat and mouse limb development. Larger expression domains in bat AER and ZPA, combined with a feedback loop caused by later re-initiation of *Fgf8* and *Shh*, contributes to lengthening in bat forelimbs (reproduced from Cooper and Sears, 2013).

The molecular basis of the general enlargement of the bat forelimb, including long bone elongation in bat wings, has been investigated using candidate gene and genomic approaches, both individually and as part of the whole limb. Candidate gene studies of the limb demonstrate that the two main signaling centers of the limb, the apical ectodermal ridge (AER) and the zone of polarizing activity (ZPA), are up to three times larger in the developing limb buds of bats than in those of mice (Cretekos *et al.*, 2007; Cooper *et al.*, 2012). The expression domain of the anterior-posterior patterning gene *Shh*, the molecular marker of the ZPA, is initially expanded in the limb buds of bats compared to those of mice (Cooper *et al.*, 2012). By the limb paddle stage (when the developing limb resembles a paddle), *Shh* expression is turned off in both bats and mice. However, *Shh* expression is later reinitiated in bats, but not in mice, due to a novel domain of *Fgf8* expression. This leads to a feedback loop (Figure 2) whereby *Fgf8* increases *Shh*, which increases *Bmp2*, which increases *Grem*, which in turn continues to prompt *Fgf8* (Hockman *et al.*, 2008). Both the prolonged expression and the larger expression domains of these genes are believed to ultimately contribute to limb enlargement and digit lengthening (Cooper *et al.*, 2012). This feedback loop also contributes to the formation of a portion of the wing membrane, which will be discussed later. Candidate gene studies have also identified a posterior expansion of the expression of *Hoxd13*, a gene with known roles in limb development, in the bat wing relative to the mouse forelimb (Chen *et al.*, 2005; Ray and Capecchi, 2008).

The bat wing has also become a frequent target of transcriptomic and genomic analyses. Comparisons of the transcriptomes of the developing wings of bats and forelimbs of mice and/or of the fore- and hind limbs of bats have revealed several genes with unique expression levels in the bat wing relative to the bat hind and mouse fore- limbs. These genes include the early limb bud marker *Meis2*, which is upregulated in the developing bat wing (Mason *et al.*, 2015), as well as all of the 5’ *HoxD* genes (*Hoxd9-13*) including *Hoxd13* (which was identified through candidate gene studies described above), *Hoxa13*, *Tbx3*, *Evx2*, and *Fam5c* (Wang *et al.*, 2014; Maier *et al.*, 2017). Studies restricted to developing digit tissues and their follow-up analyses have also identified several genes from the *Bmp* pathway, *Tbx* and *Hox* families, and others that are differentially expressed in bat wing digits. These include the genes *Tbx3*, *Tbx15*, *Bmp3*, *RGMB*, *Smad1*, *Smad4*, *Nog*, *Hoxd8*, *Hoxd9*, *Hoxa1*, *Satb1*, *Twist1*, *Tmeff2*, *Mab21l2*, and *Enpp2* (Wang *et al.*, 2010; Dai *et al.*, 2014). 

The genetic drivers underlying the patterns of bat wing gene expression described above are currently under study. Because the protein-coding regions of most patterning genes with important roles in limb development appear to be largely conserved across bats and other mammals (Chen *et al.*, 2005; Sears *et al.*, 2006; Cretekos *et al.*, 2007; Ray and Capecchi, 2008), it is likely that many if not most of the morphological specializations of the bat wing have evolved through changes in gene regulation (Petit *et al.*, 2017). 

Ten years ago, two separate studies began the search for the regulatory elements behind the unique phenotypes of the bat wing. In the first study, the authors made a genetically-modified mouse in which they replaced the limb-specific transcriptional enhancer of the *Prx1* locus with the orthologous sequence from the bat *Carollia perspicillata* (Cretekos *et al.*, 2008). The resulting mice displayed forelimb long bones that were modestly but significantly longer than controls. These results are consistent with the phenotypes of mice lacking *Prx1* function; *Prx1* null mice display defects in the limb skeleton, including shortening of multiple long bone elements of the forelimb (Martin *et al.*, 1995). The second study investigated the structure and function of a known *HoxD* limb enhancer, the Global Control Region (GCR) in bats, following up on the finding that *HoxD13* expression differs in bat and mouse forelimbs (Ray and Capecchi, 2008). Researchers identified several sequence differences in the bat and mouse GCR, and found the bat GCR capable of driving distinct expression domains in transgenic mice relative to that of other mammals. Taken together, these results support the hypothesis that changes in gene regulation contribute to the morphological specialization of the bat forelimb for powered flight.

More recently, researchers have begun to apply a systematic approach to their study of the role of regulatory evolution in the development of the bat wing. By comparing the genomes of diverse mammals and aligning observed differences to previously assembled datasets of putative mouse limb enhancers, researchers have identified 166 bat accelerated regions (BARs) that have evolved at a faster rate in bats than other mammals (Booker *et al.*, 2016). Five out of five of these putative bat enhancers displayed limb enhancer activity when tested in transgenic mice. These enhancers are located near five genes with known roles in limb development that are differentially expressed in bat wings: *Twist2*, *Spry1*, *Shh*, *Spg20*, and the *HoxD* cluster. Key among these enhancers may be BAR116, which appears to play a role in regulating the *HoxD* complex (Booker *et al.*, 2016). The role of BAR116 in the divergent development of fore- and hind limbs was further tested by Yakushiji-Kaminatsui *et al.* (2018). This study examined the CS93 sequence in chick that is believed to be orthologous to BAR116. CD93 in chick displayed similar enhancer activity to BAR116 in bat, with more expression in the fore- than hind limbs. Furthermore, the CS93/BAR116 sequence was more conserved between chicks and bats than with mice, potentially owing to the increased disparity between fore- and hind limbs in these taxa.

ChIP-Seq assays have also been performed directly on the limbs of bats. Data from these assays has been used to identify almost 3,000 potential regulatory elements that display elevated evolutionary rates in bats and are located near genes with known roles in limb development (Eckalbar *et al.*, 2016). While many of the enhancers driving wing development remain widely unknown, this research has great potential to identify genetic drivers of morphological specialization in bats and, by so doing, start filling in the genotype - to - phenotype map, a major goal of biology.

## Bat cranial structures: Variation with diet

Bats display an extraordinary amount of morphological specialization and diversity in their crania. For example, the size and shape of the ears, nose ornaments, eyes, faces, and teeth dramatically differ among species. We present a brief overview of research into the developmental basis of cranial diversity in bats, an exciting and growing area of research. For a more thorough review of how development potentially creates diversity in mammals more broadly, please see the recent review by (Usui and Tokita, 2018).

Bats display a broad array of facial lengths that are tightly linked to the functional requirements of their diets. For example, bats that feed on nectar tend to have very long faces that facilitate their penetration into flowers, while bats that feed on hard fruit have very short faces that allow them to bite with higher force (Dumont *et al.*, 2009; Nogueira *et al.*, 2009). Face shape is determined during development by coordinated growth and ossification of neural crest derived ectomesenchyme in the first pharyngeal arch (which gives rise to the mandible and maxilla) and frontonasal process (Santagati and Rijli, 2003). Facial shape can be developmentally altered by changes in the migration and number of neural crest cells during early embryonic development, and/or in maturation and growth of neural crest derived skeletal elements later in development or during postnatal growth. These skeletal elements undergo ossification either via a cartilaginous template like in the appendicular skeleton (known as endochondral ossification), or directly from the ectomesenchyme, (known as membranous ossification) (Hall, 2005). Both of these ossification processes are controlled by the transcription factor *Runx2* (Komori, 2018). The activity of *Runx2* relies on a highly conserved Runt DNA binding domain, shared by other members of the runt-like gene family, and a unique region of repeated glutamines (Q) followed by a region of repeated alanines (A). These repeated amino acids are reflected in repeated sequences within the genome. Such simple sequence repeats are more prone to mutation than many other parts of the genome and so are thought to act in fine-tuning gene expression (Kashi and King, 2006). The QA ratio in *Runx2* has been demonstrated to correlate with face length, first in domestic dogs (Fondon and Garner, 2004), and subsequently across a number of mammalian groups (Sears *et al.*, 2007; Pointer *et al.*, 2012; Ferraz *et al.*, 2018). This includes phyllostomid bats, where the Runx2 QA ratio correlates with width and length of the membranous palatine bone, which in turn influences face shape and length. In bats, a low QA ratio is found in nectarivores with long and narrow faces, while a high QA ratio is found in frugivores with short and wide faces (Ferraz *et al.*, 2018). QA repeats in the *Runx2* locus may be acting to modulate the activity of the gene, since *Runx2* acts early in bone development to promote differentiation of undifferentiated mesenchyme into osteoblasts, and later inhibits terminal differentiation of osteoblasts into osteocytes (reviewed in Komori, 2018). Therefore, changes in *Runx2* activity will affect skeletal growth, as supported by cell based expression assays demonstrating that *Runx2* activity, as determined by the expression of its target *Col10a,* increased in a linear fashion with QA ratio (Sears *et al.*, 2007). Thus it is possible that this relatively changeable domain has facilitated phyllostomid morphological evolution by altering palate dimension and thus face shape, thereby allowing for the exploitation of new diets that are dependent on face shape.

The skull is a morphologically complex structure. Geometric morphometrics enable detailed and quantitative comparison of skull shape. Recent morphometric analysis of bat skull developmental and evolutionary trajectories across species suggests that variation in phyllostomid skull morphology is driven by peramorphosis, such that development of the skull is similar across species until a basal morphology is reached and then those species with a specialized form continue developing to achieve new morphologies (Camacho *et al.*, 2019). Interestingly, bats with different morphologies seem to have subtly differing strategies for achieving peramorphosis. Generalist species and frugivores achieve their short skull form by accelerating the rate of their development, whereas nectarivores and sanguinivores diverge through a longer period of fetal and post-natal development. The hypothesis that prolonged or accelerated skull development drives morphological change in the bat crania bookends well with the hypothesized increase in *Runx2* QA tandem repeats, since extended or increased osteoblast activity facilitated by *Runx2* activity would be consistent with peramorphosis of skull bones.

The importance of *Runx2* in face length and width offers a candidate for the regulation of bat craniofacial morphology through gene regulation. Another possible candidate for this is the paired homeobox transcription factor *Pax9*. This transcription factor has a well-established role in craniofacial development, including the development of teeth (Peters *et al.*, 1998), mandibular bone (Anthwal *et al.*, 2015), the lip (Nakatomi *et al.*, 2010), and palate (Peters *et al.*, 1998; Zhou *et al.*, 2013). Phillips and colleagues (2013) found a conserved region of the 3’ untranslated region (UTR) of the *Pax9* gene containing ﻿Musashi-binding elements (MBE) in phyllostomid and ﻿vespertilionid bats. Musashi proteins are known to regulate a range of cellular processes, including stem cell fate decisions and cellular differentiation, by the inhibitory binding mRNA via MBEs. As such, MBEs may play a role in the control of *Pax9* gene expression level. The number of MBEs vary across phyllostomid bats with variable facial morphologies, but not across vespertilionids. This suggests that, at least in phyllostomids, variation in *Pax9* may contribute to the generation of facial diversity. While many other genes are known to be important in facial morphogenesis in mammals, their role in bat facial diversification remains largely unexplored. 

Another remarkable facial variation present in some bat species are facial clefts, either midline clefts or paramedian clefts, where there are two clefts on either side of the face and a remnant midline tissue. Orr and colleagues (2016) undertook an analysis of facial clefts in bats and concluded that such clefts resemble some cleft lip and palate disorders seen in human patients in that the cleft is not accompanied by other phenotypes elsewhere in the body. This is in contrast to mouse transgenic models of facial clefts such as knockouts of the *Tgf-beta* or *Fgf* signaling pathways, where many other structures of the organism are impacted. However, bats' clefts also differ in some ways from those seen in human clinical cases. Bat clefts are only found in the hard tissues of the face, whereas human clefts are found in both soft and hard tissues. Furthermore, the hard tissue clefts of bats are bridged by fibrous tissue (Orr *et al.*, 2016) that, along with the intact soft tissues, probably ensures that suckling is possible during early postnatal life. The identity of the fibrous bridging material in clefts is not known. Possibly be the remnants of the skeletogenic mesenchyme arrested before ossification, and as such clefts in bats are not due to failure in midline fusion of the orofacial primordium during embryonic development (Orr *et al.*, 2016). This hypothesis is supported by the absence of soft tissue clefts in bats. Modulation of ossification may therefore be driving the specification of these clefts in bats.

## Diversification during adaptive radiation

The concept of adaptive radiation provides a theoretical framework for explaining the taxonomic and functional diversity of organisms in response to ecological selective pressures (Simpson, 1944). In this framework, the availability of ecological opportunities, i.e. ecological niches that become available through the colonization of new environments, the extinction of competitors, or the acquisition of key innovations, is linked to rapid diversification (Yoder *et al.*, 2010). Often described as a classic example of an adaptive radiation, the acquisition of flight in bats is commonly thought to have opened up many new ecological opportunities and thereby enabled their diversification (Speakman and Racey, 1991; Simmons and Geisler, 1998; Fenton, 2010). However, while flight is likely to have played a crucial role in bats diversification, other adaptations have also likely contributed, including the specializations of the skull discussed above. In this section, we explore research in two additional systems that have likely played important roles in the adaptive radiations of bats: vision and dentition. 

## Adaptive radiations and sensory systems - an example from bat vision

Among novel phenotypic traits that are commonly thought to promote diversification (Yoder *et al.*, 2010), sensory adaptations are considered key innovations that enable access to hitherto inaccessible ecological resources. For example, trichromatic vision in primates enables fruit discrimination against a background of leaves, and echolocation in cetaceans enables orientation and hunting in low-light environments (Jacobs, 2009; Steeman *et al.*, 2009; Teeling *et al.*, 2012; Geisler *et al.*, 2014). Bats are known to possess many key sensory adaptations; perhaps the most well-known of which being their highly specialized hearing system that allows the true echolocation that helps most species navigate, forage, and/or hunt in the dark (recently reviewed in Yohe and Brand, 2018). However, research over the past 15 years has begun to highlight the importance of adaptations in other sensory structures to the evolutionary success of bats (Davies *et al.*, 2013), including variation in the sizes of the cochlea (Davies *et al.*, 2013), eyes (Heffner and Heffner, 1992; Eklöf, 2003), olfactory epithelium and olfactory bulb, as well as in the vomeronasal epithelium and accessory olfactory bulb (Bhatnagar and Smith, 2007; Smith *et al.*, 2012; Yohe *et al.*, 2017; Yohe and Brand, 2018). Of particular note, studies of the evolution of bat vision in the last two years have provided unprecedented insights into the role of sensory adaptations in bat evolution (Gutierrez *et al.*, 2018b; Kries *et al.*, 2018; Wu *et al.*, 2018; Li *et al.*, 2018; Simoes *et al.*, 2019; Sadier *et al.* 2018). Because of this, we further discuss the evolution of the visual systems of bats.

Most mammals possess three visual opsins: rhodopsin (RHO) in rods, long-wave (M/L) sensitive opsin (OPN1LW) in L-cones, and short-wave (S) sensitive opsin (OPN1SW) in S-cones (Yokoyama and Radlwimmer, 1999, Yokoyama, 2000, Zhao *et al.*, 2009). While many nocturnal animals are monochromatic (i.e., completely color‐blind as a result of only having rhodopsin and one cone opsin), others possess dichromacy and are capable of some chromatic distinctions (Peichl, 2005; Jacobs, 2009; Zhao *et al.*, 2009; Jacobs, 2012; Veilleux *et al.*, 2013). However, up to recently, our knowledge of bat vision has remained obscure. The first studies of the large eyes of flying foxes were based on microscope observations and suggested that their retinas contained only rods and no cones (Fritsch, 1911; Kolmer, 1910; Neuweiler, 1962). For a long time, 20many other species, in particular Yangochiropterans one with small eyes, were considered nearly blind. This vision started to change almost a century later when the eyes of flying foxes were reexamined, revealing that these bats also possess cones, albeit at a low cone/rod ratio, suggesting that flying foxes potentially have color vision (Jacobs, 1993) and another study tested the response of bats to different wavelengths of light (Joshi and Chandrashekaran, 1985). 

More recently, the growing availability of bat genomic data have resulted in a renewed interest in bat vision. The first study that examined the molecular aspects of opsin genes in bats investigated the S and M/L opsin genes of two species of Yinpterochiroptera and one species of Yangochiroptera sequenced at that time (Wang *et al.*, 2004). All of these species were found to possess both M/L and S opsins (Zhao *et al.*, 2009) suggesting that these species of bats, at least, are dichromatic. Then, a number of studies began to investigate opsin sequences and electrophysiological vision capabilities in bats. Using immunohistochemistry (IHC), sequencing, and electrophysiological experiments, Müller *et al.* (2009) found that both M/L and S opsins are expressed and functional in two plant-visiting phyllostomid species, and that differences in cone densities in these species are potentially linked to differences in visual acuity. A separate study in four genera of Yinpterochiroptera that also used IHCs revealed that, while all species likely possess both dim and day light vision, these genera likely vary in their ability to see in color: *Pteropus* possesses both S and M/L opsins, and thus dichromatic vision, whereas *Rousettus*, *Eidolon*, and *Epomorphorus* only express M/L opsin and, consequently, are monochromatic (Müller *et al.*, 2007). Interestingly, the authors of this study linked these differences in photoreceptor composition to the roosting preferences of these genera, as *Pteropus* is often more exposed to daylight than the others. By studying multiple species, these studies revealed that bat vision is more complex than previously thought, and established tight links between the visual capabilities of bats and their ecological niche specializations, a link that has been documented in other mammals (Douglas and Jeffery, 2014). 

Building on these discoveries, research in the last decade has investigated the relationship between ecological pressures and vision capabilities in multiple bat species. Results of these studies, most of which have been based on gene sequences alone and fairly limited taxonomic sampling, suggest that the S opsin gene is likely functional across Yangochiroptera but lost in some Yinpterochiropteran lineages (Winter *et al.*, 2003; Feller *et al.*, 2009; Zhao *et al.*, 2009; Müller *et al.*, 2009; Butz *et al.*, 2015; Gorresen *et al.*, 2015). Because inferences from amino acid sequence analyses and action spectra suggest that bat S opsins are sensitive to UV light, researchers proposed that the loss of S-opsins could have profound impacts on bat visual acuity. Accordingly, researchers hypothesized the retention of S-opsins is possibly related to the demands of visual processing in mesopic, or low-light, conditions (Zhao *et al.*, 2009), and/or plant visiting (Müller *et al.*, 2007; Feller *et al.*, 2009; Müller *et al.*, 2009; Butz *et al.*, 2015). However, the limited sampling of these studies hindered their ability to rigorously test links between vision capabilities and ecological parameters, in particular within the Yangochiroptera. 

**Figure 3 -:**
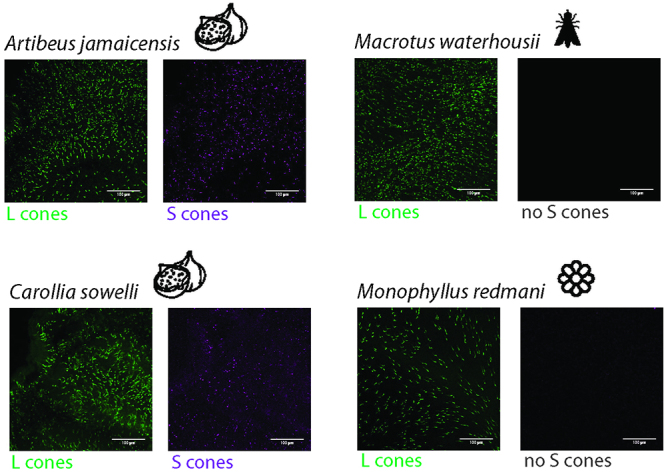
Color vision diversity in Phyllostomids (adapted from Sadier *et al.*, 2018). Cones are shown on flat-mounted retinas in four different bat species representative of the cone diversity in Phyllostomids. To visualize L opsin (green) and S opsin (magenta), flat-mounted retinas were probed with two antibodies against L and S opsins. L opsin expressing cones were found in all bats, while S opsins were only seen in fruit visiting *Carollia* and *Artibeus* species, indicating cone type and diversity vary between species occupying different dietary niches. Diet is indicated with a pictogram.

In 2018, multiple studies on bat vision featuring larger taxonomic sampling of multiple independent lineages representing diverse ecologies (e.g. blood feeding, plant-visiting species) were published. These studies revealed evidence of *OPN1SW* pseudogenization events in both Yangochiroptera and Yinpterochiroptera (Kim *et al.*, 2008; Kries *et al.*, 2018; Wu *et al.*, 2018; Li *et al.*, 2018; Simoes *et al.*, 2019), suggesting that UV vision loss is more widespread in bats than previously thought. In most of these studies the functionality of *OPN1SW* was based only on analyses of DNA and/or RNA sequence, and not on the S opsin protein. However, one study combined investigation of the conservation and expression of opsin gene sequences with the localization of M/L and S opsin proteins in 56 species of Yangochiroptera (Sadier *et al.*, 2018). Results of this study suggest that, while the DNA sequences of S opsin genes most commonly appear functional, the gene is often not translated and/or transcribed (Figure 3). This occurs in multiple independent lineages, suggesting that bat lineages take several routes to a loss of S opsin function.

To link the loss of S opsin function with ecological adaptation, studies tested for trade-offs between vision state and other sensory adaptations (Gutierrez *et al.*, 2018b; Kries *et al.*, 2018; Wu *et al.*, 2018; Li *et al.*, 2018; Simoes *et al.*, 2019), and for correlations between vision state and various ecological parameters (Wu *et al.*, 2018; Sadier *et al.*, 2018; Gutierrez *et al.*, 2018b; Simoes *et al.*, 2019). These findings revealed that UV vision is lost in many bat species, and not just in high duty cycle (HDC) echolocators (Gutierrez *et al.*, 2018b; Simoes *et al.*, 2019), and does not support the existence of a trade-off between the evolution of HDC echolocation and loss of functional S opsin. However, other studies have found some support for an association of frugivory (Sadier *et al.*, 2018) and roosting behavior (Müller *et al.*, 2007) with S opsin retention in bats, and yet others for a tradeoff between UV vision and infrared sensing in the common ancestor of vampire bats (Kries *et al.*, 2018; Li *et al.*, 2018). Bat rhodopsin has also been found to show evidence of adaptation to dim light conditions (Gutierrez *et al.*, 2018a). Very recently (Davies *et al.* 2020) found that the nectar‐feeding lineages of noctilionoids and the Stenodermatinae subfamily of fig‐eating bats exhibit molecular adaptations resulting from fine-tuning of pre-existing visual adaptations, suggesting that noctilionoids which use visual cues for identifying food and roosts and orientation were preadapted to colonized these new ecological niches. These exciting findings reveal that vision has taken a major role during bat adaptation and diversification and opens a new field of research to investigate. 

## Adaptive radiations and morphological diversification: Insights from bat teeth

Among vertebrates in general and mammals in particular, teeth are one of the most variable organs of the body, being highly diverse in terms of number, shape, and size. One of the main factors suspected of driving this diversity is dietary ecology, which is thought to have shaped dental morphology with some dental traits evolving numerous times in association with diet (Lucas, 1979, Hunter and Jernvall, 1995; Lucas and Peters, 2007). Indeed, intensive study of mammals from unrelated groups has uncovered many convergences, with highly similar dentitions characterizing distinct groups with similar diets (Evans *et al.*, 2007). 

**Figure 4 -:**
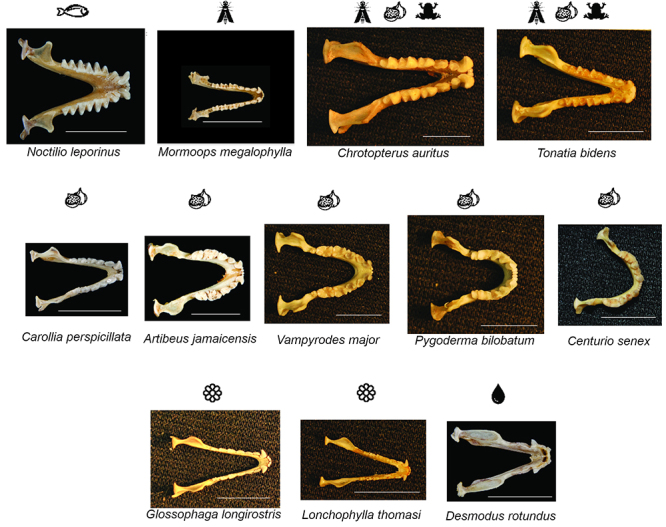
Jaw and dental diversity of Noctilionoid bats. Members of the group occupy every possible dietary niche found in bats. In line with this, jaw size and shape as well as tooth shape, size and proportion are highly variable in Noctilionoid bats. In fact, the jaw and tooth types shown represent most of the breadth of the diversity found in both bat superfamilies. Diet is indicated with a pictogram. Scale: 10 mm

Bats exhibit diverse dentitions associated with their many dietary types (Freeman, 1988; Freeman, 1998) (Figure 4). While the ancestral bat was likely insectivorous (Gunnell and Simmons, 2005), subsequent bat lineages have evolved many different diets: insectivory, carnivory, sanguivory, omnivory, nectarivory, and frugivory. Foundational work on bat teeth diversity has been primarily performed by Patricia Freeman. By studying 108 species from 78 genera from both Yinpterochiroptera and Yangochiroptera, Freeman (1998, and reviewed in Swartz *et al.*, 2003) characterized the diversity of bat teeth. She showed that the relative tooth area is highly variable between Yinpterochiroptera and Yangochiroptera, as well as within Yinpterochiroptera (particularly in carnivorous species), and linked some differences in form to diet. Insectivorous bats possess a triangular jaw with regularly spaced teeth, nectarivorous bats an elongated jaw with long and narrow teeth, and frugivorous bats a short jaw with unequal teeth. Within frugivores, stenodermatid bats have an extreme phenotype with an extremely short face and rounded jaw (Freeman, 1998). Among them, *Centurio senex* displays a striking example of the extreme phenotype of stenodermatid bats, with its extremely short face and small canines. 

Much of the subsequent research on bat dentition and diet has focused on Neotropical bats because of their incredible variation. Bats of the Noctilionidea, which includes the family Phyllostomidae (~200 species of New World leaf-nosed bats and allies within the suborder Yangochiroptera), have received particular attention because they are the only bat group in which all possible bat diets (insectivory, carnivory, piscivory, sanguivory, omnivory, nectarivory and frugivory) have been documented. This group diversified approximately 40 million years ago (Rojas *et al.*, 2013; Rossoni *et al.*, 2017) and today exhibits morphological adaptations linked to their broad dietary specializations (Monteiro and Nogueira, 2011; Dumont *et al.*, 2012; Davies *et al.*, 2013; Hayden *et al.*, 2014; Yohe *et al.*, 2015). For example, bats from this group display a diversity of biting behaviors and bite forces that facilitate their broad range of diets (Freeman, 1998; Dumont, 1999; Santana *et al.*, 2011; Dumont *et al.*, 2012). Biting behavior varies most substantially among frugivorous species of phyllostomids (Dumont, 1999). Specialized frugivores use one side of their mouth to bite their food, unspecialized frugivores use both sides, and omnivores have been shown to modulate their biting behavior in response to food hardness. Other studies have also linked the molar complexity of this group to their feeding performance (Evans *et al.*, 2007; Santana *et al.*, 2011). Taken together, these studies illuminate the diversity of diet, teeth morphology and biting behavior in bats making them an excellent group to study the diversification of an organ in relationship with its dietary ecological niche. 

Because of this, bat teeth represent a great model to study the developmental basis of morphological transitions from one diet, and tooth type, to another, a shift that occurs recurrently and rapidly in mammals. Future studies should build upon the extensive foundational work that has been done in this promising study system and investigate the developmental mechanisms underlying the evolutionary diversification of bat teeth.

## Origins of morphological novelty - Insights from bat wing membranes

In elongating their forearms and digits, bats created a scaffold for the dynamic, collapsible, and compliant tissue that comprises the membranes of the bat wing. The functional combination of membrane tension and elasticity has allowed for unparalleled flight capability in bats, which in turn has provided the basis for their remarkable diversification (Simmons, 2005; Teeling *et al.*, 2005). 

**Figure 5 -:**
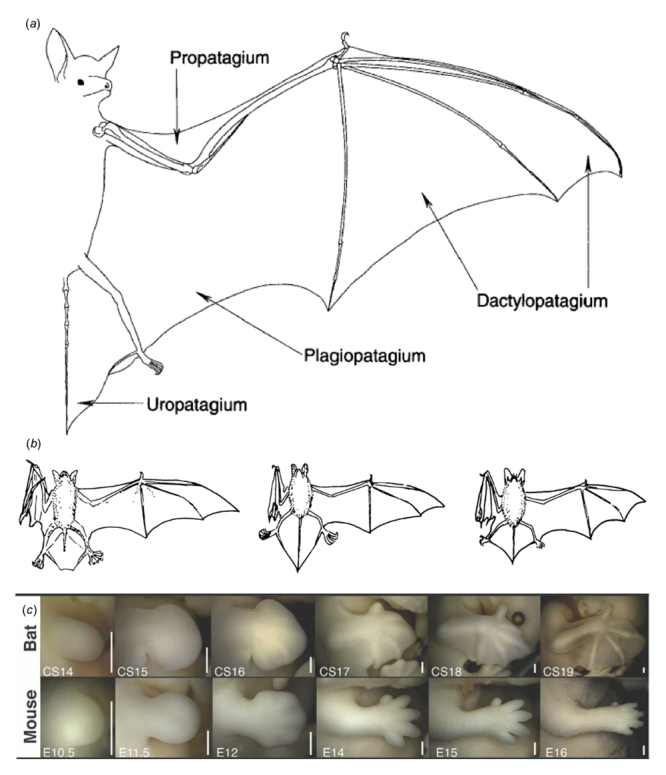
The anatomy and development of the bat wing (a) Outline of extended forelimb and wing membranes in chiropterans demonstrating the skeleton and membranes (patagia) of the wing (from Swartz *et al.*, 1996). (b) Examples of variation in size and shape of patagia (from Norberg and Rayner, 1987). (c) Early limb development in bats compared to mice showing early similarities in limb development and divergence in growth and maturation. (from Cretekos *et al.*, 2008).

The bat wing consists of three membranes (patagia): dactylopatagium, between the digits; plagiopatagium, between the fifth digit to the ankle; and the propatagium, a smaller membrane on the anterior portion of the stylopod and zeugopod (Figure 5a). Additionally, many bats also possess a membrane between the hind limbs, the uropatagium (Swartz *et al.*, 1996). Size and shape of these patagia can vary greatly between species (Figure 5b) and are often tightly correlated with both flight style and diet (Norberg, 2012), ranging from high speed in insectivores to high maneuverability in nectarivores and frugivores (Wetterer *et al.*, 2000; Gunnell and Simmons, 2012). Insectivores have long, narrow plagiopatagia that are adapted for increased speed during aerial hawking; whereas frugivores tend to have short, broad membranes that allow for increased maneuverability in cluttered vegetation environments. In general, the combined forelimb membranes generate lift, while the uropatagia provides stability and can have a functional role in catching insects during midair hunting.

Of the four membranes of the bat wing, only one, the dactylopatagium, has an obvious homology in non-bat mammals, while the other three are seemingly novel traits. The dactylopatagium is also the only bat wing membrane for which the underlying developmental origins have been reasonably resolved. Mice, and most other mammals, undergo interdigital tissue regression following condensation of the digits (Figure 5c). In bats, the continued presence of *Fgf8* combined with the *Bmp* inhibiting effects of *Grem* prevent the activation of the apoptotic pathway that would otherwise degrade the interdigital tissues and lead to freed digits (Weatherbee *et al.*, 2006; Cooper and Sears, 2013). The result is the retention of interdigital tissues into the adult as the dactylopatagium (Figure 5c).

Beyond the developmental origins of the dactylopatagium, the developmental processes leading to the formation of the patagia and their subsequent diversification into a multitude of sizes and shapes remain largely unknown. One potential clue to the drivers of these phenomena was the discovery by Tokita *et al.* (2012) that expression of *Fgf10* continues in the anterior-proximal portion of the bat forelimb mesenchyme, long after expression in other taxa (e.g., *Mus*, *Monodelphis*, and *Gallus*) had ceased. However, while *Fgf10* signaling is active in areas where future wing membranes develop, its expression is restricted to the area immediately adjacent to the wing musculature. This suggests that some other mechanism(s) are responsible for the outgrowth and formation of the membrane itself. 

As truly novel structures, the patagia (plagio-, pro-, and uro- patagia) beyond the dactylopatagia lack any known homology within mammals. These novel patagia play a significant role in chiropteran flight abilities, and so to comprehensively understand how flight has evolved in bats we need to understand the origin and diversification of these novel membranes. 

## Conclusions

Through a series of adaptive radiations, bats have evolved to be highly diverse. This diversity makes bats a model system for addressing biological questions on topics including biomechanics, morphological evolution, longevity, developmental evolution, sensory system adaptations, and many others. In this review, we have provided an overview of how evolution and development have molded some of the most unique morphological specializations of bats. 

The elongated forelimb bones that support bat wings display higher growth rates than those of other mammals. This accelerated growth is preceded by formation of ZPA’s and AER’s in bat forelimbs that are three times larger than those of mice, coupled with changes in the associated *Shh* and *Fgf8* feedback loop. Transcriptomic and genomic analyses have also revealed significant upregulation of *HoxD* genes and several other affiliated pathways in bat forelimbs relative to the forelimbs of other mammals. Evidence is starting to accumulate that changes in gene regulation strongly contribute to these differences in gene expression and the highly specialized forelimb structures of bats. 

The cranial features of bats (ears, nose ornaments, and faces) can vary dramatically from species to species. Facial length is tightly correlated with diet type and activity of the *Runx2* transcription factor, and regulation of *Pax9* expression has been linked to bat facial diversity as well. Alterations in overall skull shape among bats have been shown to be accomplished through the acceleration and extension of development. Meanwhile, the facial clefting that some bats exhibit is likely due to modulation of ossification, leaving soft tissues unaffected. 

Bat sensory systems, especially vision, have also been found to vary much more from species to species than previously assumed. Until fairly recently, most bat species were thought to have monochromatic vision, possessing rods and perhaps only L- or S- opsins. However, newer studies have shown that surprising numbers of bat species possess both L- and S-cones, and thus likely have dichromatic vision. This phenotype has been linked to ecological specializations (diet and roosting habits). Several lineages have also been found to have lost UV vision through multiple routes of S- opsin loss of function, including post-transcriptional processes. 

Bats have evolved many feeding specializations, and the dentition of different groups has been tightly linked with diet type. Both tooth area and proportion are highly variable among bat species, with molar complexity and biting behavior also varying substantially from bat to bat and species to species. Because of their diversity and strong ties to diet, bat teeth have the potential to be a great model for understanding how changes in ecological niche lead to diversification of biological structures. 

In addition to their numerous specializations in existing morphological structures, bats also possess truly novel morphological structures in their plagio-, pro-, and uro- patagia. While the developmental processes that have shaped the evolution of another wing membrane, the dactylopatagium, have been explored, the processes driving the formation of these novel membranes remain unknown. Given the importance of these membranes to powered flight in bats, future study of the development of these novel membranes is critical to our understanding of the evolution of powered flight and has the potential to provide insights into the processes behind the evolution of novel traits, an outstanding question in evolutionary biology. 

Each of the areas discussed here have ample room for future research, and there is much more work to be done to uncover the full range of developmental mechanisms underlying the specializations and diversity of bat morphology. Time and time again, bats have proven to be a model system for study of the developmental basis of morphological specialization, evolutionary novelty, and adaptive radiation. We look forward to the many answers bats have yet to reveal. 
